# Butler-Matrix Beamspace Front-Ends for Massive MIMO: Architecture, Loss Budget, and Capacity Impact

**DOI:** 10.3390/s25237170

**Published:** 2025-11-24

**Authors:** Felipe Vico, Jose F. Monserrat, Yiqun Ge

**Affiliations:** 1 Universitat Politècnica de València (UPV)—iTEAM, 46022 Valencia, Spain; jomondel@upv.es; 2 Huawei Technologies Company Ltd., Shenzhen 518129, China; yiqun.ge@huawei.com

**Keywords:** Butler matrix, beamforming network, beamspace, massive MIMO, CSI feedback, switching matrix, winner-takes-all, insertion loss, capacity, 6G

## Abstract

Massive Multiple-Input Multiple-Output (MIMO) systems with hundreds or thousands of antenna elements are fundamental to next-generation wireless networks, promising unprecedented spectral efficiency through spatial multiplexing and beamforming. However, the computational burden of channel state information (CSI) acquisition and processing scales dramatically with array size, creating a critical bottleneck for practical deployments. While previous works demonstrated that Fast Fourier Transform (FFT)-based beamspace processing can exploit the inherent angular sparsity of wireless channels to compress CSI feedback, the digital implementation requires intensive computations that become prohibitive for ultra-large arrays. This paper presents an analog alternative using Butler matrices—passive beamforming networks that realize the Discrete Fourier Transform in hardware—combined with RF switching circuits to select only dominant angular components. We provide a comprehensive analysis of Butler matrix architectures for arrays up to 32 × 32 elements, characterizing insertion losses across different technologies (microstrip, substrate-integrated waveguide, and waveguide) and operating frequencies (10–30 GHz). The proposed system incorporates parallel power sensing with Winner-Take-All circuits for sub-microsecond beam selection, drastically reducing the number of active RF chains. Full-wave simulations and capacity evaluations at 12 and 30 GHz demonstrate that the Butler-based approach achieves comparable performance to FFT methods while offering significant advantages in power consumption and processing latency. For a 256 × 256 array, FFT computation requires 0.36 ms compared to near-instantaneous analog processing, making Butler matrices particularly attractive for real-time massive MIMO systems. These findings establish Butler matrix front-ends as a practical pathway toward scalable, energy-efficient beamspace processing in 6G networks.

## 1. Introduction

Large-scale or ultra-large-scale Multiple-Input Multiple-Output (MIMO) systems are fundamental to the evolution of wireless communications toward Sixth Generation (6G) networks. By deploying hundreds or thousands of antenna elements at the Base Station (BS), these systems enable highly directive beamforming that significantly improves User Equipment (UE) signal quality and overall spectral efficiency. A particularly compelling application is Tera-bps MIMO (T-MIMO) operating in the 10–14 GHz range and millimeter Wave (mmW) bands, where the availability of wider bandwidth channels promises unprecedented data rates. However, this potential comes with substantial challenges: the overhead associated with Channel State Information (CSI) acquisition, feedback, and processing scales dramatically with both the number of antenna elements and the channel bandwidth, threatening to overwhelm the computational resources of practical systems.

Recent work by the authors in [1] addressed this challenge by demonstrating that the per-subcarrier channel matrix H exhibits significant sparsity when transformed to the angular domain via a two-dimensional Discrete Fourier Transform (DFT). This angular sparsity—arising from the physics of electromagnetic propagation where only a few dominant paths carry most of the signal energy—enables dramatic compression of the channel representation. By operating in this reduced-dimensional beamspace, both CSI feedback overhead and the computational complexity of detection/precoding can be substantially reduced without sacrificing system performance.

Despite these advantages, the digital FFT-based approach faces practical limitations when scaled to ultra-large arrays. The computational burden of performing repeated 2D FFTs across hundreds of antenna ports and multiple subcarriers introduces significant latency and power consumption, potentially negating the benefits of massive MIMO in real-time applications. For instance, a single 2D FFT for a 256 × 256 array requires approximately 0.36 ms of processing time, consuming over one-third of the typical 1 ms update interval in modern wireless systems.

This paper presents an analog realization of beamspace processing using Butler matrices—passive microwave networks that implement the DFT directly in the RF domain. By replacing digital FFT computations with Butler matrix transformations, followed by low-complexity RF switching to retain only the dominant angular components, we achieve the same dimensionality reduction with near-zero latency and significantly reduced power consumption. The Butler matrix approach is particularly attractive for ultra-large arrays where digital processing becomes prohibitive, offering a practical pathway toward scalable massive MIMO implementations.

### 1.1. Contributions

- Comprehensive design and analysis of 1D and 2D Butler matrices for massive MIMO, including their mathematical equivalence to the DFT beamspace basis and practical implementation considerations up to 32 × 32 arrays.
- Detailed characterization of insertion loss scaling with array size *N* and implementation technology (microstrip, SIW, waveguide), explicitly separating the fundamental $10\log_{10}N$ power division from technology-dependent excess losses.
- Development of a complete analog beamspace architecture incorporating scalable switching fabrics (including Beneš networks) and parallel power-sensing circuits with Winner-Take-All selection, achieving sub-μs beam identification.
- End-to-end system evaluation including protocol design and capacity analysis at 12 GHz and 30 GHz for both symmetric and asymmetric array configurations, demonstrating comparable performance to FFT-based methods with substantial reductions in power consumption and latency.

### 1.2. Paper Organization

Section 2 reviews related work on hybrid beamforming and Butler matrix implementations. Section 3 presents the massive MIMO system model. Section 4 and Section 5 develop the Butler matrix architecture, including detailed loss analysis and 1D/2D designs. Section 6, Section 7 and Section 8 describe the switching network, power measurement with WTA circuits, and the complete system protocol. Power consumption comparisons and capacity results are presented in Section 9 and Section 10, respectively, followed by Section 11.

## 2. Related Works

Hybrid beamforming has emerged as a key enabler for mmW massive MIMO (mMIMO) in Fifth Generation (5G) networks, providing a compromise between performance, hardware cost, and energy efficiency. A comprehensive survey in [2] analyzes hybrid beamforming methodologies from architectural and system-modeling perspectives, including the composition of digital and analog beamforming matrices, antenna deployment strategies, and applications in heterogeneous networks. It also highlights open challenges such as channel state information (CSI) acquisition, analog/digital optimization, and resource management.

Architectural variants of hybrid beamforming are commonly classified as fully connected or sub-connected (partially connected). Fully connected structures achieve higher beamforming flexibility at the expense of hardware complexity and loss, while sub-connected structures reduce circuit footprint and power consumption but operate in a constrained subspace, limiting achievable capacity [2]. Beyond general architectures, innovative schemes such as the hybrid Fourier-transform approach in [3] employ Butler matrices for analog azimuthal beamforming while reserving digital control for elevation, thereby reducing search complexity. Related works [4,5] explore similar hybrid digital–analog trade-offs.

The physical implementation of Butler matrices has been widely studied. A recent review [6] summarizes advances in circuit-level realizations, including compact two-dimensional Butler matrices. While Blass matrices provide alternative beamforming networks with simpler layouts, they incur larger circuit footprints. Beyond these matrix-based networks, lens-based beamformers such as Rotman lenses realize fixed beams through passive true-time-delay structures, mapping different input ports to distinct angular directions. Rotman lenses provide inherently wideband beamforming and have been demonstrated at microwave and mmWave frequencies, but they typically occupy a larger volume than planar Butler networks and require careful design to control aberrations and beam squint. Compared to these alternatives, Butler matrices offer a compact, approximately orthogonal beamspace basis that is straightforward to integrate in planar technologies, which motivates their use as the analog front-end in the present work. Despite extensive theoretical exploration, practical Butler-based implementations remain limited. Notable contributions include the investigation in [7] and the compact switched network antenna of [8], which integrates a 4×4 Butler matrix with a patch array at 28 GHz for 5G NR (n258/n261). This design achieves four switchable beams with high gain (∼11.5 dBi) in a compact footprint, while also demonstrating compliance with safety standards, confirming its applicability in wearable mmWave devices.

The use of Butler matrices for angle-domain processing of MIMO channels was initially proposed in [9,10,11]. These works emphasized the advantages of low-cost analog transforms for spatial sparsity exploitation, though experiments were restricted to 4×4 MIMO systems. Scaling such methods to larger arrays remains critical for next-generation deployments. More recently, digital frameworks have been proposed to harness angular-domain sparsity in ultra-large-scale MIMO. The patent in [12] introduces an FFT-based compression technique with selective thresholding of dominant beams, enabling efficient channel characterization while reducing complexity and estimation overheads.

Together, these studies illustrate the spectrum of approaches to angular-domain processing and hybrid beamforming: from theoretical surveys and architectural comparisons to concrete Butler-matrix implementations and FFT-based digital compression. They highlight both the promise of analog transforms in reducing RF-chain requirements and the complementary role of digital FFT-based strategies in managing CSI feedback and computational load in future large-scale MIMO systems.

## 3. Massive MIMO Channel Model and Angular Sparsity

For each OFDM subcarrier, the downlink of a massive MIMO system can be modeled as

(1) y=Hx+w,

where $x\in C^{M}$ is the transmitted signal across *M* antenna ports, $y\in C^{N}$ is the received signal at *N* ports, and w denotes additive noise. The channel matrix $H\in C^{N\times M}$ encapsulates the propagation effects between the base station and the user equipment. Accurate knowledge of H is essential for precoding and detection, but explicit feedback of all its entries quickly becomes infeasible as *M* and *N* scale to hundreds or thousands of ports.

**Figure 1:**
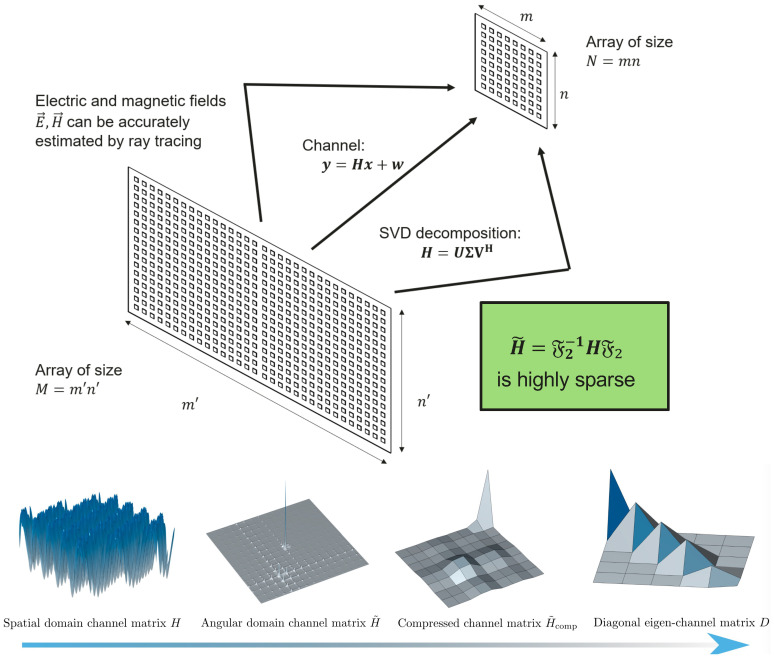
Ray-tracing view of a massive MIMO channel: only a few dominant propagation paths contribute significant energy. This sparsity motivates compression of the channel matrix via FFT into a compact angular-domain representation.

From a physical perspective, at frequencies in the 10–30 GHz range, electromagnetic propagation is dominated by only a few significant rays—typically a direct line-of-sight path and a limited set of first-order reflections. Higher-order interactions exist but contribute little energy. This *sparse multipath structure* can be captured by ray-tracing models, where the channel is expressed as a superposition of a small number of plane waves aligned with the dominant angular directions. Figure 1 illustrates this picture: a handful of propagation paths determine most of the channel energy.

The mathematical implication is that, after applying a two-dimensional Discrete Fourier Transform (2D DFT) across the antenna array, the transformed channel

(2) $$\tilde{H}=F_{2}^{-1}HF_{2}$$

is *highly sparse*: only a small set of angular bins contains significant values, corresponding to the physical rays in the propagation environment. This angular sparsity suggests that channel feedback and processing can be greatly simplified by operating in the Fourier (beamspace) domain, where compression and reduced-dimension detection/precoding are naturally enabled.

## 4. Channel Compression via Butler Matrices

TThe proposed approach mirrors the Fast Fourier Transform (FFT)-based beamspace method in [1], but the transformation is carried out entirely in the analog domain. This shift offers significant power savings: the two-dimensional Fourier transform is realized through cascaded Butler matrices, while a switching network selects only the dominant angular components. After this selection, only the reduced set of outputs is connected to the Analog-to-Digital (A/D) Radio Frequency (RF) chains. As a result, the number of active RF chains scales with the effective angular support of the channel rather than with the total number of antenna elements, yielding substantial reductions in both hardware complexity and energy consumption.

**Figure 2:**
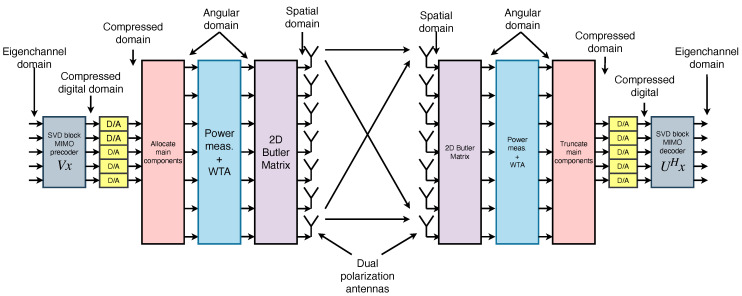
Block diagram of channel compression using Butler matrices. The FFT blocks are replaced by analog Butler networks, followed by a switching stage to retain only dominant angular components.

Figure 2 shows the full block diagram of this architecture, where the digital FFT blocks at the transmitter and receiver are replaced by analog Butler matrices. The figure highlights the successive stages of angular transformation, beam selection through switching, and subsequent low-dimensional digital processing.

## 5. Butler Matrix Design and Implementation

### 5.1. Introduction to Butler Matrices

A Butler matrix is a passive microwave network widely used as a beamforming network (BFN) in antenna arrays. Its function is to map one or more input signals to multiple output ports, such that each output corresponds to a distinct fixed beam direction. The structure is typically an N×N network, with *N* usually chosen as a power of two [13,14].

The circuit is built from interconnected 3dB hybrid couplers, which split and combine signals with precise amplitude and phase relationships, and fixed phase shifters, which impose additional $45^{{}^{\circ}}$ or $90^{{}^{\circ}}$ shifts along specific paths. Together, these elements generate a set of orthogonal phase progressions across the outputs, effectively realizing a discrete Fourier transform in hardware.

This arrangement ensures that each input is evenly distributed and phase-adjusted to form well-defined beams, enabling simultaneous multi-beam operation with minimal complexity. Because the Butler matrix is passive, reciprocal, and compact, it has found extensive use in radar front-ends, satellite payloads, and multi-beam wireless communication systems where efficient analog beamforming is essential.

### 5.2. Design and Validation of Butler Matrices

The Butler matrices were designed and validated using the commercial full-wave solver CST Studio Suite 2025 (Dassault Systèmes). Each network follows the classical configuration described in [15], implemented here in microstrip technology. Alternative planar platforms such as coplanar waveguide (CPW) or grounded coplanar waveguide (GCPW) could also be adopted. The substrate parameters for the hybrid couplers are summarized in Table 1, while those for the phase shifters appear in Table 2.

**Figure 3:**
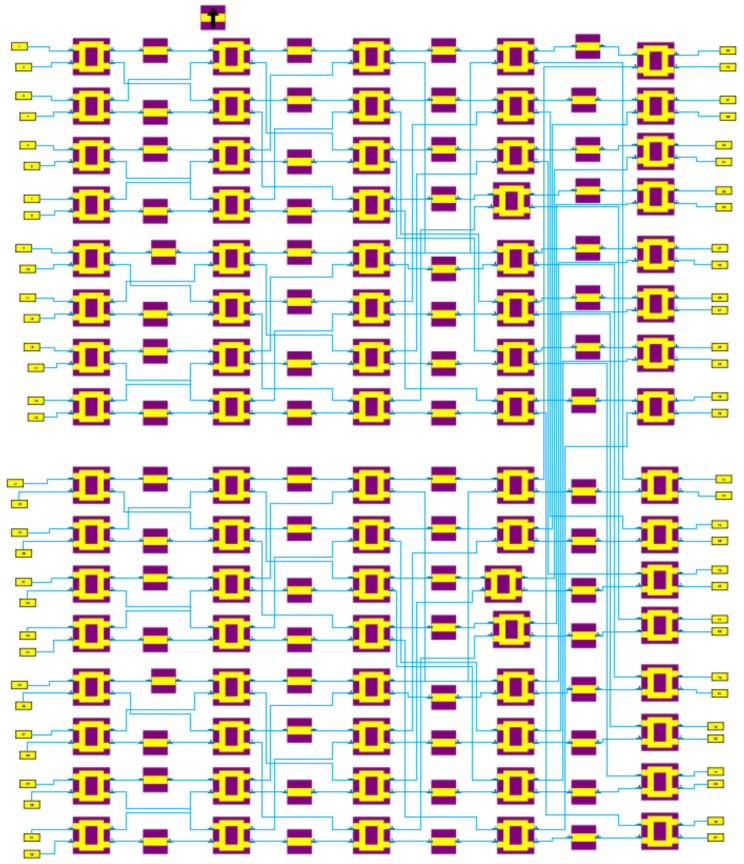
Design of the 32×32 Butler matrix in CST.

**Figure 4:**
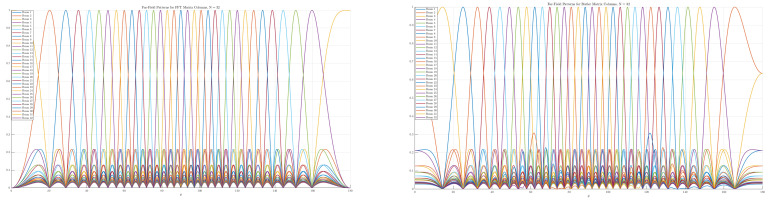
Far-field beams for N=32: FFT vs. Butler matrix weights.

The final implementation considered in this work is the 32×32 Butler matrix, whose CST design is shown in Figure 3. Validation was carried out by comparing the far-field beams obtained from the Butler weights with those from an FFT-based implementation. As shown in Figure 4, the two approaches produce nearly identical orthogonal beam patterns, confirming that the analog transform reproduces the angular decomposition predicted by the FFT model.

In summary, the design and validation of the 32×32 Butler matrix confirm that this classical architecture effectively decomposes the angular domain into orthogonal beams, while providing a practical analog alternative to digital FFT-based beamspace transforms.

It is important to emphasize that the CST model of the 32×32 Butler matrix is used here primarily to validate the angular decomposition and to illustrate the feasibility of large planar implementations. The insertion-loss budgets and technology comparisons employed in Section 5.5 and Section 9 are not taken directly from this single CST model, but from a compilation of measured and simulated Butler prototypes reported in the literature (see Table 3). These works include realizations in microstrip, substrate-integrated waveguide, and hollow waveguide technologies at frequencies between 5 and 32 GHz, and provide realistic excess losses under fabricated conditions. A detailed hardware realization and over-the-air characterization of the specific 32×32 design considered here is left for future work.

### 5.3. 2D Butler Matrix Configuration

**Figure 5:**
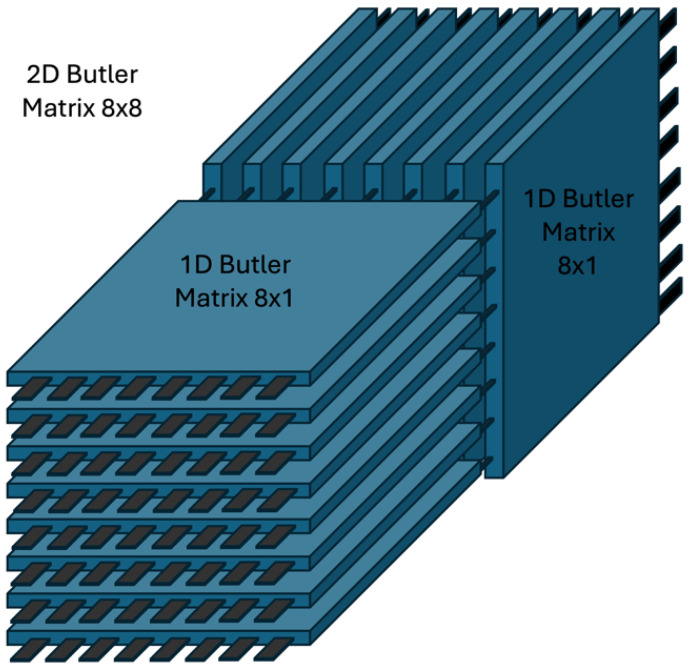
Conceptual 2D Butler configuration: row- and column-wise 1D Butler stages compose a separable 2D transform.

A two-dimensional (2D) Butler matrix for a uniform planar array is obtained by cascading 1D Butler stages along rows and columns, yielding a separable (Kronecker) realization of the 2D Fourier transform. The concept is sketched in Figure 5.

### 5.4. Depth Analysis of 2D Butler Matrices

The longitudinal depth of a Butler network scales with the number of cascade stages, which grows as $\log_{2}N$ for an N×N array. A practical footprint estimate assigns about $0.3\lambda_{g}$ per hybrid and includes meandered phase shifters, yielding an effective stage length of ≈ $0.6\lambda_{g}$ (with $\lambda_{g}$ the guided wavelength set by the substrate’s effective permittivity). Using a high-$\varepsilon_{r}$ substrate (e.g., $\varepsilon_{r}=7.5$) further compacts the layout.

For a 1D Butler matrix,

(3) $$L_{\mathrm{depth}}^{1D}\approx 0.6\;\lambda_{g}\;\log_{2}N,$$

and for a 2D structure (row+column cascades),

(4) $$L_{\mathrm{depth}}^{2D}\approx 1.2\;\lambda_{g}\;\log_{2}N.$$

Table 4 lists representative depths and array footprints at 10 and 30 GHz for $\varepsilon_{r}=7.5$.

As *N* increases, the module aspect ratio (array plus 2D Butler) decreases, which is favorable for integrating very large arrays with compact front-end depth.

### 5.5. Insertion Losses in Butler Matrices

An ideal *N*-port Butler matrix evenly divides power with phase progression, so each output receives 1/N of the input; the fundamental *N*-way split is $10\log_{10}\;N$ dB (e.g., 6 dB for N=4, 9 dB for N=8). Practical implementations incur additional *excess* insertion loss from finite coupler/directive performance, conductor and dielectric loss, impedance mismatch, crossovers, and longer interconnects.

Complexity grows with *N*—for example, a 16×16 network may require on the order of 32 hybrids, 24 phase shifters, and ∼60 crossovers—so excess loss accumulates with the number of stages and routing density. The hybrid count scales roughly as $\left(N/2\right)\log_{2}N$, while crossover count grows super-linearly, tightening amplitude/phase balance tolerances.

Two reporting conventions exist: (i) total insertion loss (including the fundamental split), and (ii) excess insertion loss beyond the split. We focus on excess loss, as it isolates technology- and design-dependent penalties. At 10–30 GHz, the microstrip/CPW tends to be lossier than SIW, while the hollow waveguide is the lowest loss but bulky and costly.

#### 5.5.1. Insertion-Loss Trend with Increasing *N*

**4 × 4.** Lowest complexity; typical excess ∼1–2 dB at X/Ku/Ka in well-optimized planar designs (SIW at 30 GHz ∼2 dB; microstrip at Ku ∼1 dB). Low-loss coaxial/waveguide forms can reach <0.6 dB at C-band.

**8 × 8.** Excess commonly ∼2–4 dB at 10–30 GHz; SIW at 28–31 GHz ∼2 dB; broadband microstrip 2–5.6 dB; waveguide <0.4 dB (X-band).

**16 × 16.** Ideal split is 12 dB; measured excess around 2.5–3 dB at 9–11 GHz (multilayer microstrip); SIW (cascaded 4×4) ∼1.43 dB (sim.) at 28–32 GHz; high-frequency measured data remain limited.

#### 5.5.2. Technology Comparison

**Microstrip.** Simple and compact integration with planar arrays; higher conductor/dielectric/radiation losses, especially as *f* and *N* rise (typ. ∼2–5 dB excess near 28 GHz).

**CPW.** Easy crossovers/integration; radiation and mode conversion can elevate loss above microstrip at mmWave.

**SIW.** Quasi-waveguide with lower loss than microstrip; typical excess ∼2 dB at 28–30 GHz; tighter fabrication and bandwidth constraints.

**Waveguide.** Lowest loss (<1 dB excess for 8×8 at X-band); bulky and expensive; scaling large *N* is challenging.

**Other.** Stripline, SICL, and gap-waveguide can balance loss and integration; metamaterial loading can reduce phase error but remains technology-dependent.

The excess insertion losses used in this work for power and capacity evaluations are drawn from these published designs rather than from our own CST model alone. As a result, conductor and dielectric losses, radiation, mismatch, and typical fabrication effects are already implicit in the reported figures. For larger Butler sizes, the trends observed in these references are extrapolated using the known scaling of the number of hybrids, phase shifters, and crossovers with *N*, providing realistic but technology-agnostic benchmarks for the subsequent system-level analysis.

From a modeling standpoint, the excess losses used in the power and capacity evaluations in Section 9 and Section 10 are thus taken from a combination of full-wave analyses and experimental measurements reported in the cited references, rather than from a single in-house EM model. Conductor and dielectric losses, as well as radiation and mismatch, are implicitly included in those published figures. Our CST design of the 32×32 Butler matrix is primarily employed to verify beam orthogonality and to confirm that the microstrip implementation behaves as an approximate DFT, while the loss numbers for 4×4, 8×8, and 16×16 networks follow the empirical trends observed in the literature. These trends are then extrapolated to larger sizes using the known scaling of hybrid, phase-shifter, and crossover counts with *N*, providing realistic but technology-agnostic benchmarks for very large arrays.

Manufacturing tolerances further impact both loss and phase accuracy in practical realizations. Hybrid coupler amplitude and phase imbalance, finite isolation in crossovers, connector and transition parasitics, and line-width variations introduce additional attenuation and phase errors that accumulate with the number of stages. As *N* grows, these non-idealities lead to increased beam-to-beam leakage and a moderate degradation of orthogonality. For this reason, the results reported in this work for arrays beyond 32×32 should be interpreted as trend indicators based on realistic but idealized component behavior. The detailed design of a specific hardware prototype for a given technology node would require a dedicated EM analysis including tolerances and layout constraints in order to refine the loss and phase-error budgets.

### 5.6. Two-Dimensional Far-Field Radiation Patterns of Butler Matrices

**Figure 6:**
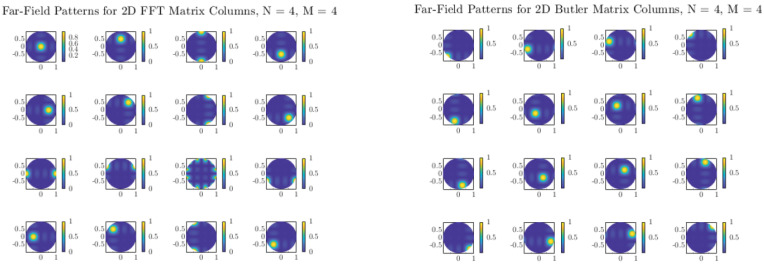
Two-dimensional far-field radiation patterns for N=4: comparison of FFT and Butler matrix weights.

**Figure 7:**
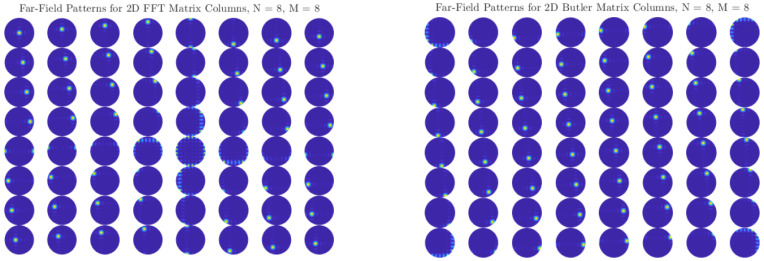
Two-dimensional far-field radiation patterns for N=8: comparison of FFT and Butler matrix weights.

Figure 6 and Figure 7 show the two-dimensional (2D) far-field radiation patterns obtained from arrays steered with either FFT-based digital weights or with the Butler matrices designed in Section 5.2. In both cases, the results reveal a systematic decomposition of the angular domain into orthogonal beams, confirming that the Butler network accurately replicates the discrete Fourier transform (DFT) basis in the analog domain.

For small arrays (e.g., N=4), the angular coverage is coarse, and each beam spans a relatively wide lobe. As *N* increases to 8, 16, or 32, the beamwidth narrows and the number of resolvable angular directions increases, providing finer granularity in spatial selectivity. This trend highlights a key advantage of Butler-based beamforming: without additional digital processing, the analog network directly produces well-defined, nearly orthogonal beams that partition the entire angular space.

Comparisons with FFT-based digital beamforming show excellent agreement. Minor differences between the two approaches can arise from implementation effects such as insertion losses, finite phase-shifter accuracy, or coupling imbalance in hybrids, but these do not significantly alter the orthogonality or coverage of the beams. Importantly, the analog Butler implementation achieves this result passively, requiring no high-speed multiplications or digital hardware scaling with array size.

The close match between FFT and Butler radiation patterns across all tested array sizes confirms that Butler matrices can serve as practical front-end beamformers in massive MIMO systems, offering the same angular sparsity exploitation as digital FFT beamspace methods, but at substantially reduced complexity and power consumption.

## 6. Switching Matrix

The switching stage plays a central role in enabling dimensionality reduction after the Butler transformation. While the Butler matrix transforms the spatial domain into an angular-domain representation, the switching network selects only the most relevant angular channels, thereby minimizing the number of active RF chains without sacrificing performance.

In general terms, a switching matrix refers to a reconfigurable hardware structure that routes multiple inputs to multiple outputs according to a specified selection rule. Such systems are common in telecommunications, data center networks, and RF front-end architectures. In our context, the requirement is more stringent: the switches must operate at microwave and millimeter-wave frequencies, offering low insertion loss, high isolation, and fast reconfiguration to support dynamic beam selection.

**Figure 8:**
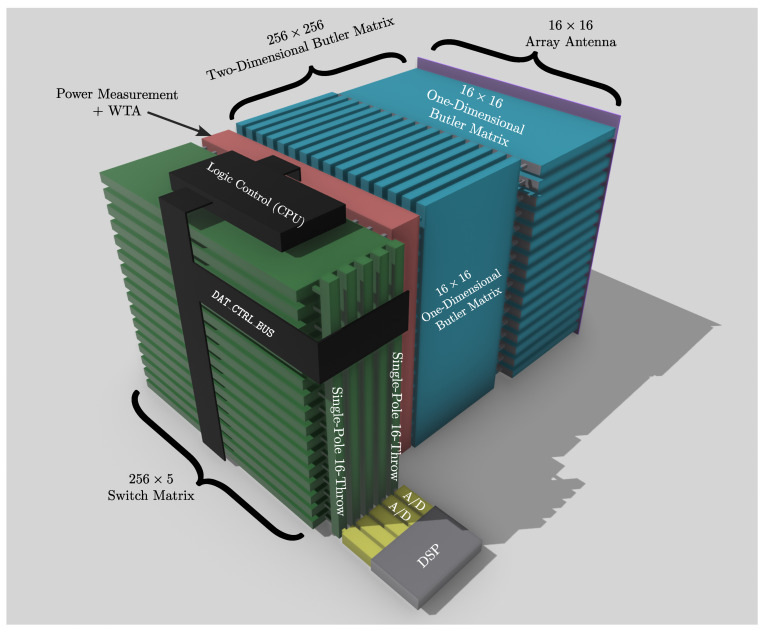
Two-dimensional Butler matrix combined with a switching stage for a 16×16 antenna array. The full 256 angular outputs are pruned down to m=5 dominant beams through the switching network.

Figure 8 shows the complete architecture, where a two-dimensional (2D) Butler matrix is cascaded with a switching matrix. The depicted case corresponds to a 16×16 antenna array, which, after the 2D Butler transform, results in 256 angular-domain ports. To reduce complexity, only a small subset of beams is retained—in this example, m=5 dominant angular channels.

The construction of the 2D Butler matrix follows a separable row–column decomposition. The first stage applies 16 one-dimensional Butler matrices across the array rows, and the second stage applies another 16 Butler matrices along the columns, resulting in a 256×256 overall transformation. The switching matrix is then tasked with selecting a small set of angular ports from this large pool.

A practical implementation can be realized using 16 parallel 16×m crossbar switches combined with *m* SP16T (single-pole, 16-throw) devices. This structure enables efficient fan-in/fan-out while keeping per-device complexity manageable. The outcome is a system where only the *m* most energetic angular beams are preserved, significantly lowering RF-chain count, power consumption, and digital workload, while retaining nearly all the channel capacity provided by the full array.

### 6.1. Comparison of Device Properties

Table 5 summarizes representative RF switches available on the market. The comparison highlights key metrics such as switch type, number of ports, operating frequency range, insertion loss, and switching speed. These parameters are critical when selecting devices for high-frequency beamspace front-ends.

Although these devices provide excellent coverage across microwave and millimeter-wave bands, none of them individually satisfy the port-count requirements of large Butler-based beamspace architectures. In particular, the maximum number of input–output ports remains well below what is needed to scale to arrays with hundreds of angular channels.

**Figure 9:**
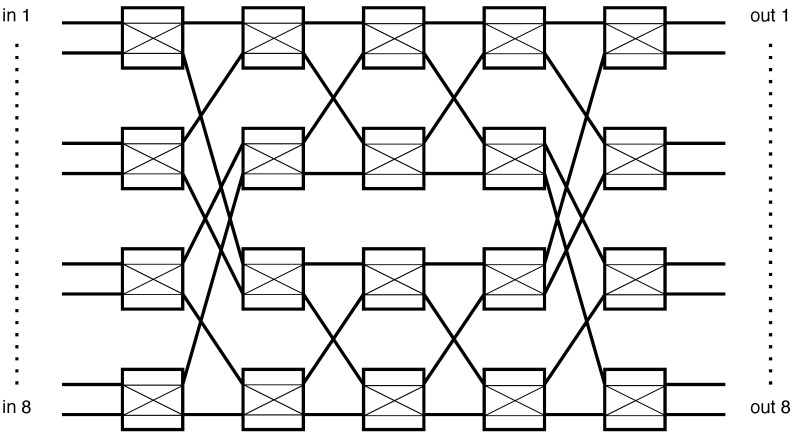
Construction of an 8×8 switching matrix using cascaded 2×2 switches in a Benes topology.

Two complementary strategies can be adopted to overcome this limitation:

- **Custom crossbar designs:** A large monolithic crossbar provides flexible interconnection but is challenging to bias and control at high speed. Increasing the number of DC bias points generally worsens switching latency, making this solution less scalable.
- **Benes-network architectures:** A more practical alternative is to interconnect multiple smaller switches in a recursive topology. As illustrated in Figure 9, the Benes network expands a modest 2×2 element into a full 8×8 or larger non-blocking fabric. Crucially, switching delay remains essentially that of the underlying device, since routing decisions are distributed across multiple short paths.

For a quantitative perspective, an N×N Benes network constructed from 2×2 switch elements has a path depth of $2\log_{2}N-1$ stages. Using the specifications in Table 5, a conservative per-stage insertion loss on the order of 1–2 dB and isolation in the 30–40 dB range can be assumed for state-of-the-art mmWave switches. This suggests that 64×64 and 128×128 fabrics remain feasible with total path losses of a few to several dB, whereas scaling to 256×256 ports would require careful co-design, on-wafer or multi-chip-module integration, and possibly custom switch technology to keep cumulative loss and crosstalk within acceptable limits.

In conclusion, a distributed Bènes-based design represents a scalable solution for massive MIMO beamspace processing: it achieves the required port count while preserving nanosecond-scale switching performance inherited from the underlying 2×2 devices. In the power and capacity evaluations of Section 9 and Section 10, we therefore model the switching stage as contributing a representative per-path excess loss in the range of 1–3 dB, rather than as a fully detailed 256×256 Benes implementation. The corresponding results should be interpreted as illustrating the potential of scalable switching fabrics built from small, low-loss building blocks, while the detailed RF design and experimental validation of ultra-large Benes networks at 30 GHz remain open research problems.

### 6.2. Switching Matrix and Main Components

Once the channel matrix H is transformed into the angular domain by the FFT-equivalent Butler matrix, the resulting $\tilde{H}$ is sparse: only a handful of beams contain significant energy. To fully exploit this property, an additional compression step is performed through an RF switching matrix that selects only the dominant angular components.

The switching block operates directly in the analog domain. Incoming beamspace signals are first power-sensed, and their levels are fed into a simple logic-control circuit. Based on a threshold or winner-takes-all criterion, the control logic enables the switches associated with the strongest beams while discarding the rest. The selected signals are then routed from the relevant input ports to a reduced set of outputs, which are connected to A/D RF chains.

Figure 8 shows an example of this architecture for a 16×16 array. A two-dimensional Butler transform generates 256 angular channels, from which only m=5 are retained by the switching fabric. The hardware can be implemented using a combination of 16 local 16×m crossbars and *m* SP16T devices, providing an effective compromise between scalability and loss.

By pruning the beamspace representation in this manner, the dimensionality of the channel is reduced without sacrificing the dominant paths. This significantly lowers hardware complexity and ADC power consumption while retaining the essential channel information for detection and precoding.

## 7. Power Measurement and Selection of Dominant Components

Efficient operation of the switching matrix requires identifying the strongest angular components in the beamspace channel. This step is critical because only a small subset of Butler outputs carries most of the energy. To achieve this, we first measure the power at all output ports of the Butler matrix and then select the dominant beams for further processing. The goal is to accomplish this task quickly and with minimal additional loss, so that system capacity and timing are not degraded.

### 7.1. Power Measurement

The first stage consists of extracting a small fraction of the signal at each Butler output using a directional coupler. This sample is fed into a simplified power detector circuit that provides a DC voltage proportional to the RF power. A common implementation uses a diode-capacitor envelope detector, although commercial integrated solutions offer higher performance and temperature stability.

**Figure 10:**
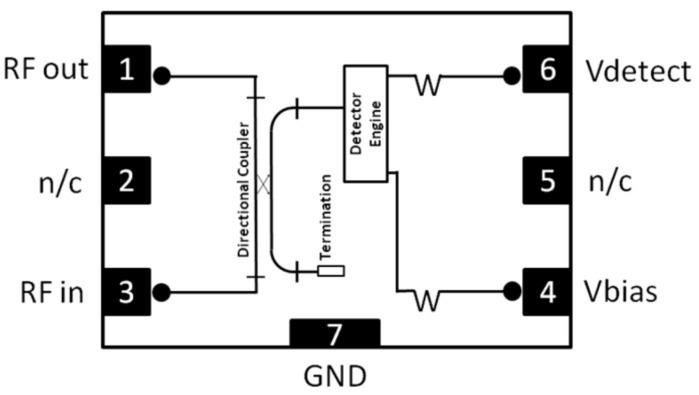
Schematic of the MACP-010572 power detector from MACOM.

An example is the MACOM MACP-010572 device (Figure 10), which integrates both a directional coupler and a detector. Its main features are:

- Integrated directional coupler with broadband response (6–18 GHz).
- Low insertion loss: 0.27 dB at 12 GHz.
- Minimum detectable power: −16 dBm at 12 GHz.

Other commercial alternatives include:

- **Analog Devices ADL5920**—Broadband directional bridge with dual RMS detectors.
- **Qorvo QPF4518**—Wi-Fi front-end module with integrated RF power detector.
- **MACOM temperature-compensated detectors**—Designed for radar and aerospace.

This measurement stage must be implemented in parallel across all Butler outputs to avoid sequential delays. The resulting voltages are then compared to determine which ports correspond to the dominant angular directions.

### 7.2. Winner-Take-All Circuits

To identify the strongest beams among *N* outputs, a large-scale comparison of the measured power levels is required. A direct digital approach would be too slow and power-hungry, so analog *Winner-Take-All* (WTA) circuits provide an attractive solution.

WTA circuits are competitive networks that select the largest among multiple inputs, asserting a “winner” output while suppressing the others. They are widely used in neural networks, vision sensors, and analog front-ends where fast selection is required. For our application, a WTA circuit ensures that only the ports with maximum detected power are routed through the switching fabric.

Two main design styles are common:

- **Voltage-mode WTAs:** Inputs are treated as voltages and compared through cascaded amplifiers or comparators. Feedback ensures that only one output remains active. These designs emphasize resolution (detecting small differences in input power) but their delay tends to grow with the number of inputs *N* due to loading.
- **Current-mode WTAs:** Inputs are represented as currents competing on a shared node. The cell with the largest input current dominates, forcing others off. These designs achieve fast response with regenerative feedback, though often at higher static power consumption.

Well-designed WTA circuits can achieve decision times scaling as O(logN) with circuit area scaling as O(N), making them suitable for real-time beam selection in massive MIMO systems [24].

### 7.3. Reported Performance

Table 6 summarizes timing and resolution results from published CMOS WTA circuits across different process technologies. Reported delays range from hundreds of microseconds in early designs to tens of nanoseconds in modern submicron implementations, demonstrating that nanosecond-scale selection is feasible for arrays with thousands of beams.

### 7.4. Summary

In summary, parallel power measurement combined with fast WTA selection enables the identification of the dominant angular beams within tens of nanoseconds. This makes it possible to dynamically reconfigure the switching matrix in real time, preserving only the most relevant channel components. As a result, the effective channel dimensionality is reduced with negligible overhead, improving the overall efficiency of the massive MIMO front-end.

**Figure 11:**
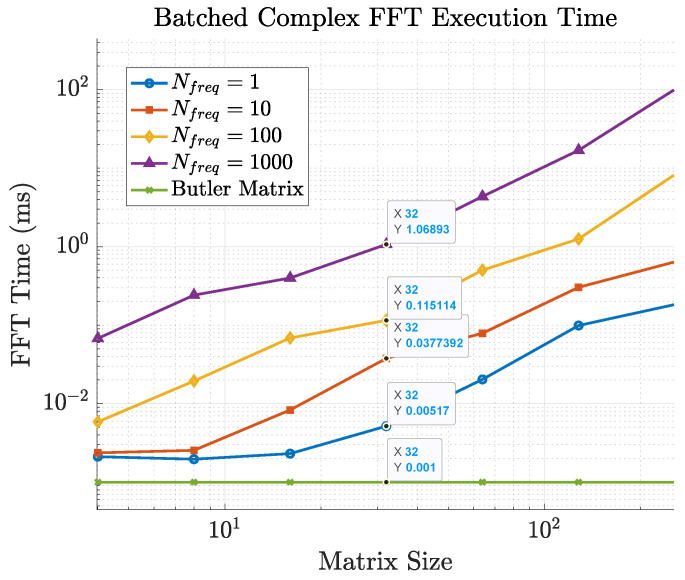
FFT computation time versus array size $n^{2}$ for different numbers of frequency channels. The Butler matrix selection+switch delay remains nearly constant and significantly lower.

The timing and sensitivity assumptions used in this section are not based on a full circuit-level co-simulation of the detector, WTA network, and biasing circuitry at 30 GHz, but on existing component data. In particular, the RF-to-DC conversion and detector response are inferred from datasheet specifications of integrated coupler–detector devices such as MACP-010572, ADL5920, and similar components, while the WTA decision times are taken from the CMOS implementations summarized in Table 6, which report selection delays ranging from tens of nanoseconds to a few microseconds for input counts up to several thousand. Combined with the RF switch on/off and settling times in Table 5, these figures support the sub-microsecond beam-selection times discussed in this work, which should therefore be viewed as realistic but not yet experimentally demonstrated targets for an integrated front-end. Even if detector RC constraints or circuit-level non-idealities were to increase the total selection time into the low-microsecond range, the analog Butler-based approach would still remain substantially faster than repeated large 2D FFT computations for the array sizes considered (cf. Figure 11). A detailed circuit implementation and co-simulation of the detector–WTA chain is left as an important direction for future work.

## 8. Full Description of the Protocol

**Figure 12:**
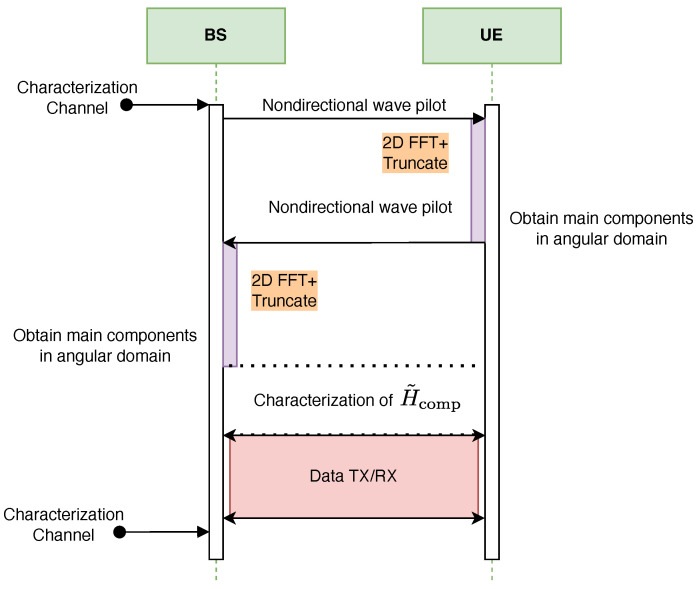
Full cycle of T-MIMO channel characterization using FFT, followed by data transmission.

**Figure 13:**
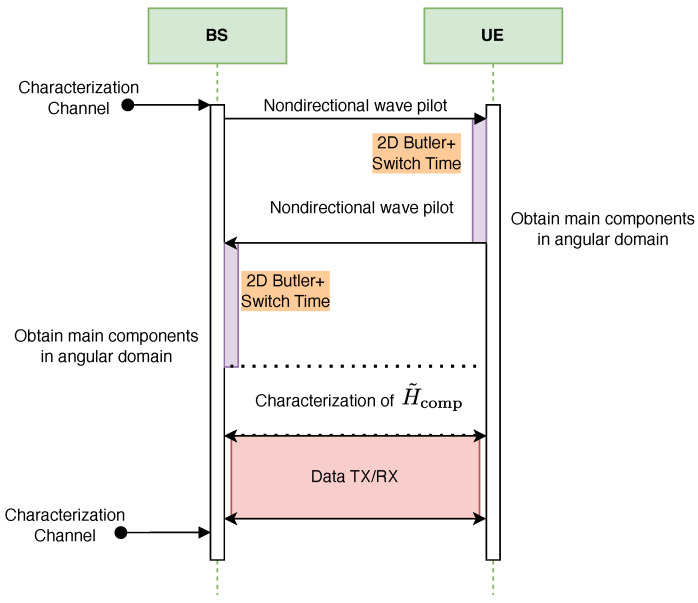
Full cycle of T-MIMO channel characterization using Butler matrices, followed by data transmission.

Figure 12 and Figure 13 illustrate the proposed protocol for full characterization of the T-MIMO channel on a single frequency subcarrier. The procedure is organized into three main stages. First, the dominant angular components of the channel are extracted at both transmitter (TX) and receiver (RX). Second, the reduced-dimension channel matrix ${\tilde{H}}_{\mathrm{comp}}$ is estimated. Finally, the third stage consists of data transmission and reception using the compressed representation.

The dominant angular components can be obtained either through a numerical FFT or via an analog Butler matrix. In the FFT-based method, performance is limited by CPU computation time, whereas in the Butler-based method the bottleneck is the switching delay of the RF circuits.

**Figure 14:**
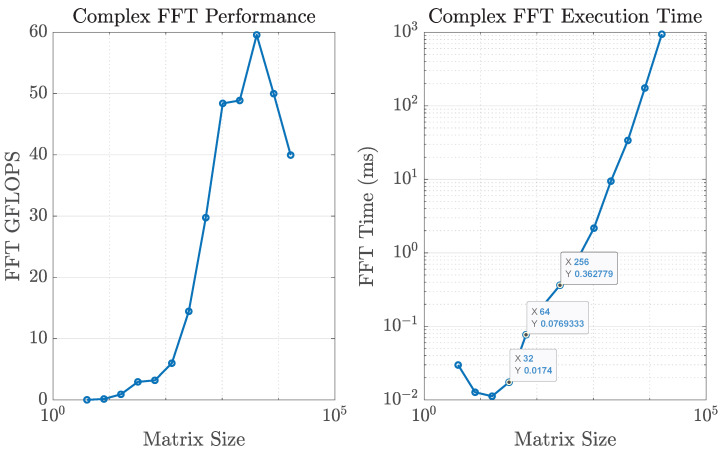
CPU time required for a single 2D FFT as a function of array size $n^{2}$.

To illustrate the trade-off, Figure 14 reports the CPU time required for 2D FFT computations of different sizes on a standard laptop. For an array of size 32×32, a single FFT requires only 17.4 μs, confirming that the numerical FFT approach is practical for moderate array sizes. However, at 256×256 the runtime increases to 0.36 ms, which represents more than one third of the 1 ms time budget available for channel updates in a typical frame. At this scale, Butler matrices become more attractive, since their processing delay is determined by switching hardware and remains nearly constant regardless of array size.

**Figure 15:**
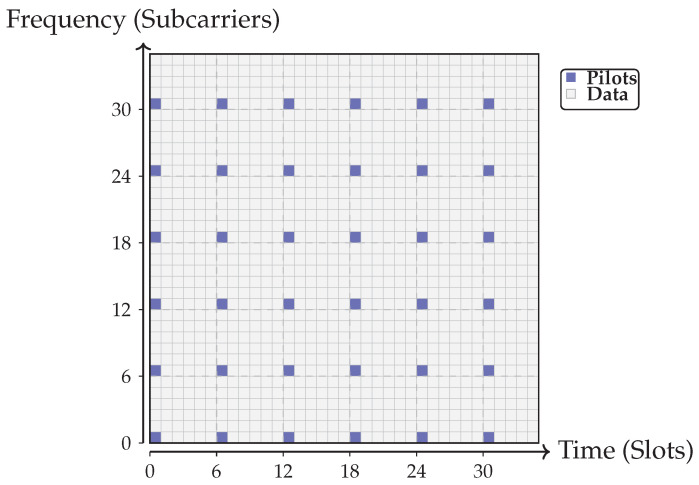
Pilot-aided estimation grid for massive MIMO systems. Black squares mark channel estimation points, while white squares correspond to data symbols (as in Figure 12 and Figure 13).

The analysis so far assumes estimation on a single frequency channel. In practice, estimation is not performed on every subcarrier but instead on a subset, followed by interpolation across frequency. In current 4G/5G systems, the sampling grid is relatively dense, as illustrated in Figure 15. Black squares indicate pilot positions where the channel matrix is explicitly estimated; white squares correspond to data transmission and reception.

The computational burden of FFT-based estimation grows both with array size and with the number of frequency points. Figure 11 compares the cost of 2D FFTs for different array sizes when $N_{\mathrm{freq}}=1,10,100,$ and 1000 frequency samples are used. The runtime scales linearly with $N_{\mathrm{freq}}$, quickly making the FFT solution impractical. In contrast, the delay of the Butler-based approach—dominated by selection and switching time—remains nearly constant with both array size and number of subcarriers, thanks to its analog nature. This makes Butler matrices particularly advantageous for ultra-large-scale arrays and wideband operation.

### 8.1. Training Overhead and Impact on Spectral Efficiency

The protocol in Figure 12 and Figure 13 consists of two main phases: (i) a training and beam-selection phase, where the dominant angular components are identified at both the transmitter and the receiver, and (ii) a data phase, where transmission takes place over the selected reduced-dimensional channel. In this subsection we quantify the training overhead associated with the beam-selection phase and discuss how it scales with array size and affects the overall spectral efficiency.

We consider a block-fading model in which the channel is approximately constant over a coherence interval of duration $T_{\mathrm{coh}}$, corresponding to $N_{\mathrm{coh}}$ OFDM symbols,

(5) $$N_{\mathrm{coh}}=\frac{T_{\mathrm{coh}}}{T_{\mathrm{sym}}},$$

where $T_{\mathrm{sym}}$ denotes the OFDM symbol duration (including cyclic prefix). Within each coherence block, $N_{p}$ symbols are devoted to beamspace training and selection, and the remaining $N_{d}=N_{\mathrm{coh}}-N_{p}$ symbols are used for data transmission.

Let $N_{B,\mathrm{TX}}$ and $N_{B,\mathrm{RX}}$ denote the total number of available beams at the transmitter and receiver, respectively, after the 2D Butler transform (e.g., $N_{B,\mathrm{TX}}=N_{B,\mathrm{RX}}=N^{2}$ for an N×N array). A straightforward beam-search procedure can be described as follows. First, the transmitter sequentially excites a subset $B_{\mathrm{TX}}\subseteq \left\{1,\ldots ,N_{B,\mathrm{TX}}\right\}$ of $|B_{\mathrm{TX}}|$ beams with pilot symbols, while the receiver observes the corresponding Butler outputs and identifies the strongest receive beams. This requires $|B_{\mathrm{TX}}|$ pilot symbols if one symbol per transmit beam is used. A similar procedure can be applied in the reverse direction (uplink), or the receiver-side beams can be inferred from downlink measurements, depending on whether FDD or TDD operation is assumed.

In the simplest case of exhaustive beam sweeping, $B_{\mathrm{TX}}$ and $B_{\mathrm{RX}}$ contain all available beams, so that

(6) $$N_{p}\propto N_{B,\mathrm{TX}}+N_{B,\mathrm{RX}},$$

and the training overhead grows linearly with the number of beams, which in turn scales with the array size. However, the angular sparsity of the channel renders such exhaustive scans unnecessary in many scenarios. In practice, hierarchical or multi-resolution beam-search procedures are often employed: the transmitter first sweeps a coarse set of wide beams to localize the dominant angular region, and only a small subset of fine beams within this region is probed in a second stage. In this case, the number of probed beams $|B_{\mathrm{TX}}|$ and $|B_{\mathrm{RX}}|$ grows much more slowly than the nominal number of beams $N_{B,\mathrm{TX}}$ and $N_{B,\mathrm{RX}}$, and can be kept approximately constant as the array aperture increases, provided that the number of dominant paths remains small.

The impact of training overhead on spectral efficiency can be captured by

(7) $$\eta_{\mathrm{eff}}=\left(1-\frac{N_{p}}{N_{\mathrm{coh}}}\right)\eta_{\mathrm{data}},$$

where $\eta_{\mathrm{data}}$ denotes the achievable spectral efficiency (e.g., capacity in bit/s/Hz) during the data phase, as reported in Section 10. The factor in parentheses represents the fraction of time available for data transmission within each coherence block after accounting for beamspace training and selection. Equation (7) shows that the key quantity governing the overall efficiency is the ratio $N_{p}/N_{\mathrm{coh}}$: for slowly varying channels (large $N_{\mathrm{coh}}$) or for efficient beam-search procedures (small $N_{p}$), the overhead becomes negligible.

It is important to emphasize that, for a given training strategy (i.e., for a fixed choice of $B_{\mathrm{TX}}$, $B_{\mathrm{RX}}$, and pilot structure), the number of pilot symbols $N_{p}$ is essentially the same for FFT-based and Butler-based beamspace processing. In both cases, the same set of angular beams must be probed in order to locate the dominant components. The difference between the two approaches lies in how the pilot observations are processed: in the FFT-based method, each set of antenna-domain pilot samples must be transformed via a 2D FFT, whereas in the Butler-based method the angular decomposition is performed directly in the analog domain, and only power detection and Winner-Take-All selection are required. As a consequence, the training overhead $N_{p}$ and the corresponding factor $\left(1-N_{p}/N_{\mathrm{coh}}\right)$ in (7) are comparable for both architectures, but the computational latency and power consumption associated with processing the pilot symbols are significantly lower in the Butler-based implementation.

In summary, beamspace training and selection introduce a well-defined overhead that can be expressed in terms of the number of pilot symbols per coherence block and that scales with the number of probed beams. The proposed Butler-based front-end does not fundamentally change this overhead relative to a digital FFT-based implementation, but it does reduce the processing burden associated with each training instance, which is especially beneficial in scenarios with short coherence times or large numbers of frequency channels.

### 8.2. Impact of Time-Varying Channels and User Mobility

The protocol described above assumes a quasi-static channel over the duration of training, beam selection, and data transmission on a given subcarrier. In practice, user mobility leads to time-varying channels whose coherence time must be compared against the latency of the beamspace selection process. In this subsection we discuss this aspect and quantify typical coherence times at the operating frequencies of interest.

A standard measure of channel variability is the Doppler spread $f_{D}=v/\lambda$, where *v* is the user speed and $\lambda =c/f_{c}$ is the carrier wavelength at frequency $f_{c}$. A commonly used approximation for the coherence time $T_{c}$ of a narrowband fading channel is

(8) $$T_{c}\approx \frac{0.423}{f_{D}}=\frac{0.423\;\lambda }{v},$$

which provides the time interval over which the channel can be regarded as approximately constant. Table 7 reports representative values of $T_{c}$ for carrier frequencies of 12 and 30 GHz and for three typical speed regimes: pedestrian (3 km/h), urban vehicular (50 km/h), and highway (120 km/h).

These values can be directly contrasted with the latency associated with beamspace selection. For the digital FFT-based approach, the dominant contribution at large array sizes is the computation of repeated 2D FFTs across the antenna ports and subcarriers. As shown in Figure 14, a single 256×256 2D FFT requires approximately 0.36 ms on a general-purpose CPU. When multiple frequency points are processed (Figure 11), the FFT-based approach can consume a non-negligible fraction of the coherence interval, especially at mmWave frequencies and high user speeds. For instance, at 30 GHz and v=120 km/h the coherence time is on the order of 0.13 ms, which is shorter than the 0.36 ms required for a single large 2D FFT in our baseline implementation. This illustrates that, in highly mobile mmWave scenarios, purely digital beamspace selection based on repeated large FFTs must rely on dedicated hardware accelerators or reduced FFT sizes to remain compatible with the channel dynamics.

In contrast, the proposed Butler-based architecture performs the angular transform entirely in the analog domain, so its latency is dominated by the time needed to measure the power at the Butler outputs, execute the Winner-Take-All (WTA) selection, and reconfigure the RF switching network. As discussed in Section 7, reported WTA circuits with hundreds to thousands of inputs achieve decision times from a few tens of nanoseconds up to the microsecond range, while state-of-the-art RF switches from Table 5 exhibit on/off and settling times between approximately 20 and 100 ns. These latency figures are derived by combining published timings for WTA circuits and RF switches, as well as detector response times, rather than from measurements on a dedicated over-the-air testbed. They therefore represent realistic but still model-based estimates of the achievable selection delay in an integrated Butler-based front-end. Even when these contributions are added, the total beam-selection latency remains well below 1 μs, i.e., two to three orders of magnitude smaller than the coherence times in Table 7 for the considered speeds and carrier frequencies.

From a system perspective, this means that for pedestrian and moderate vehicular speeds at 12–30 GHz the proposed Butler-based scheme can readily track the channel evolution within each coherence block: beam selection and reconfiguration occupy only a negligible fraction of $T_{c}$, and the channel can be treated as approximately constant over the subsequent data interval. At higher speeds and mmWave frequencies (e.g., 30 GHz and 120 km/h), the coherence time becomes comparable to or smaller than typical frame durations in current systems, which makes any beamspace method more challenging. In these regimes, the very low latency of the analog Butler front-end is particularly advantageous, as it allows more frequent reselection of the dominant beams or the use of short training bursts without consuming a significant portion of the coherence interval.

Finally, we note that the performance results reported in Section 10 correspond to a block-fading model in which the channel is assumed constant over the duration of training and data transmission on each subcarrier. The discussion above indicates that this assumption is well justified for the targeted operating regimes, especially when using the Butler-based implementation. Extending the analysis to explicitly model fast time variations and to design predictive or tracking mechanisms for the dominant beams is an interesting topic for future work.

## 9. Power Consumption Comparison

This section compares the power consumption of the FFT-based method with that of the Butler matrix approach. The analysis considers both the reduction in the number of active transceivers achieved by the Butler architecture and the additional insertion losses introduced by its analog implementation.

**Figure 16:**
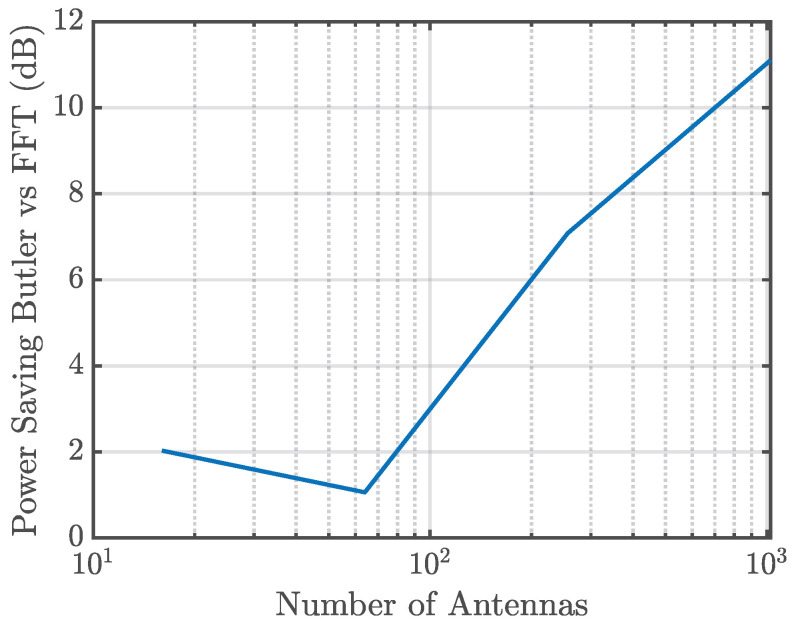
Comparison of total power consumption: Butler matrix vs. FFT-based method.

Figure 16 illustrates the relative power savings obtained with the Butler matrix:

The power savings are modeled as:

(9) $$P_{\mathrm{saving}}\left(\mathrm{dB}\right)=2\left(L_{\mathrm{Butler}}\left(\mathrm{dB}\right)+L_{\mathrm{Switching}}\left(\mathrm{dB}\right)\right)-10\log_{10}\;\left(\frac{N_{\mathrm{active}}}{N_{\mathrm{total}}}\right),$$

where $L_{\mathrm{Butler}}$ denotes the excess insertion loss of the Butler matrix (see Section 5.5) and $L_{\mathrm{Switching}}$ represents the losses of the switching stage (see Section 6). The factor of two accounts for both the transmit (Tx) and receive (Rx) signal paths. The last term captures the power savings due to the drastic reduction in the number of active RF chains, made possible by the dimensionality reduction performed in the analog domain.

The results confirm that power savings grow with array size and remain positive over the range considered (from 16 to 1024 antennas), demonstrating the efficiency of the Butler matrix solution for large-scale MIMO arrays.

### 9.1. Additional Considerations

The above model provides a first-order estimate of power savings, but higher-order effects—particularly the relative overhead of digital versus analog processing—must also be considered.

The FFT-based solution requires intensive digital processing, including high-speed FFT computation, frequent memory access, and high-frequency clocking, all of which significantly contribute to power consumption at large array sizes. In contrast, the Butler matrix operates entirely in the analog domain, avoiding these digital overheads.

This distinction suggests that the Butler-based scheme offers even greater energy-efficiency advantages when system-level digital signal processing (DSP) power is included in the analysis. In practice, for very large arrays, the DSP burden of FFT operations can dominate the overall power budget, making the analog Butler matrix particularly attractive for energy-constrained or large-scale deployments.

### 9.2. Effect of Analog Losses on SNR and Receiver Sensitivity

The power-consumption analysis in the previous subsection focuses on the reduction in the number of active RF chains and on the associated savings in front-end and digital processing power. In this subsection we explicitly relate the excess insertion losses of the analog Butler and switching stages to receiver sensitivity and SNR degradation, with particular emphasis on operation at 30 GHz.

From a link-budget perspective, any passive loss *L* (in linear units, L>1) that appears in the RF chain reduces the available signal power by a factor 1/L. On the transmit side this corresponds to a decrease in the effective radiated power, while on the receive side it manifests as a degradation of the receiver noise figure and, consequently, of the SNR. When the insertion loss occurs *before* the low-noise amplifier (LNA), its contribution to the overall noise figure can be obtained with Friis’ formula. If $L_{\mathrm{pre}}$ denotes the total pre-LNA loss (linear) and $F_{\mathrm{LNA}}$ and $G_{\mathrm{LNA}}$ are the noise factor and gain of the LNA, respectively, the cascaded noise factor is

(10) $$F_{\mathrm{tot}}=F_{1}+\frac{F_{2}-1}{G_{1}}=L_{\mathrm{pre}}+\frac{F_{\mathrm{LNA}}-1}{1/L_{\mathrm{pre}}}=L_{\mathrm{pre}}\;F_{\mathrm{LNA}}.$$

In dB, this can be written as

(11) $${\mathrm{NF}}_{\mathrm{tot},\mathrm{dB}}\approx L_{\mathrm{pre},\mathrm{dB}}+{\mathrm{NF}}_{\mathrm{LNA},\mathrm{dB}},$$

so that each dB of pre-LNA loss increases the effective noise figure by approximately 1 dB and reduces the SNR at the detector by the same amount.

In the proposed architecture, the Butler matrix and the switching network can be placed either before or after the LNA, depending on the integration strategy. A conservative configuration at very high frequencies places the analog network before the LNA, in which case its excess loss directly impacts the noise figure. As a representative example at 30 GHz, consider a Butler implementation with an excess insertion loss of $L_{\mathrm{Butler}}=2$ dB and a switching stage with $L_{\mathrm{Switch}}=1$ dB on the receive side. The total pre-LNA loss is then $L_{\mathrm{pre},\mathrm{dB}}=3$ dB, corresponding to a linear factor $L_{\mathrm{pre}}\approx 2$. If the LNA has ${\mathrm{NF}}_{\mathrm{LNA}}=2$ dB, the overall noise figure becomes

(12) $${\mathrm{NF}}_{\mathrm{tot},\mathrm{dB}}\approx 3\;\mathrm{dB}+2\;\mathrm{dB}=5\;\mathrm{dB},$$

implying a 3 dB SNR degradation relative to a reference receiver without the analog beamspace network. When the same Butler and switching structures are also present on the transmit side, the end-to-end link budget is reduced by approximately 6 dB (3 dB at the transmitter and 3 dB at the receiver) if the transmit power and LNA gain are kept unchanged.

At 30 GHz, where the free-space path loss is already significantly higher than at 12 GHz due to the smaller wavelength, this additional 6 dB penalty must be carefully considered. For example, a 6 dB reduction in link budget corresponds to either halving the maximum coverage distance (for a path-loss exponent close to 4) or, equivalently, reducing the available SNR margin by 6 dB for a fixed coverage radius. In practice, part of this penalty can be compensated by the highly directive beams enabled by large arrays, as well as by placing LNAs as close as possible to the antenna elements so that the Butler network and switches operate after a first gain stage. In that case, the excess losses mainly reduce the signal level entering the subsequent stages but have a much smaller impact on the overall noise figure.

It is also important to contrast this SNR penalty with the power savings achieved by the Butler-based front-end. The reduction in the number of active RF chains significantly lowers the total power consumed by LNAs, mixers, local oscillators, and data converters. For a massive array, turning off a large fraction of RF chains can lead to power savings that outweigh the effect of a few dB of analog loss on the link budget, particularly in small-cell and indoor deployments where coverage constraints are less stringent than in wide-area macro-cell scenarios.

The capacity curves reported in Section 10 are obtained under a block-fading model in which the Butler and FFT transforms are treated as unitary (i.e., without explicitly embedding the analog losses of the front-end). In this setting, the effect of the Butler network is to compress the channel into a lower-dimensional beamspace while preserving the total channel energy. The additional analysis presented here shows how realistic insertion losses would translate into an SNR offset that can be incorporated into the capacity expressions by simply shifting the operating SNR by $L_{\mathrm{pre},\mathrm{dB}}$ on the receive side (and by $L_{\mathrm{pre},\mathrm{dB}}$ on the transmit side if applicable). This separation allows the impact of angular-domain compression and that of front-end losses to be assessed independently, and it highlights the design trade-off between SNR degradation and RF-chain power savings in Butler-based beamspace architectures.

## 10. Capacity Results Comparison

In our previous work [1], we reported capacity results obtained with the FFT-based approach under both simulated and realistic channel conditions. That study addressed a wide range of scenarios, including indoor and outdoor environments, and operating frequencies such as 12 GHz and the mmW bands. Both symmetric and asymmetric antenna configurations were examined, with different array sizes.

In this paper, we extend that analysis by comparing the FFT-based method with the proposed Butler matrix implementation, in order to assess their relative performance and quantify the benefits of reducing computational complexity.

**Figure 17:**
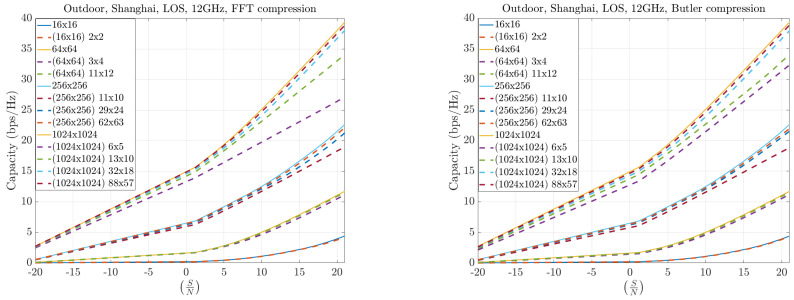
Capacity results at 12 GHz for symmetric MIMO configurations.

**Figure 18:**
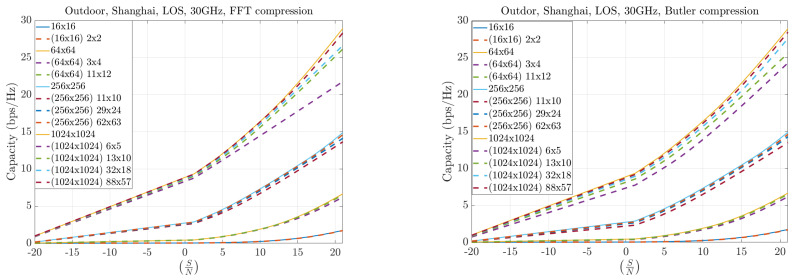
Capacity results at 30 GHz for symmetric MIMO configurations.

Figure 17 and Figure 18 present results for symmetric MIMO array configurations at 12 and 30 GHz, respectively, across different compression levels. The solid curves represent the full Singular Value Decomposition (SVD) decomposition, which serves as the theoretical upper bound. The dashed curves correspond to the practical throughput obtained with compression: the left plots show the FFT-based method, while the right plots show the Butler matrix solution. The results indicate only marginal differences between the two methods, both of which remain close to the theoretical bound. Furthermore, as the compression level increases, both approaches converge toward the ideal capacity, particularly for larger arrays.

A key observation is that larger arrays consistently outperform smaller ones, even under high compression. This demonstrates that capacity gains due to increased aperture dominate the performance trade-off, validating the effectiveness of large-scale arrays for high-throughput systems.

**Figure 19:**
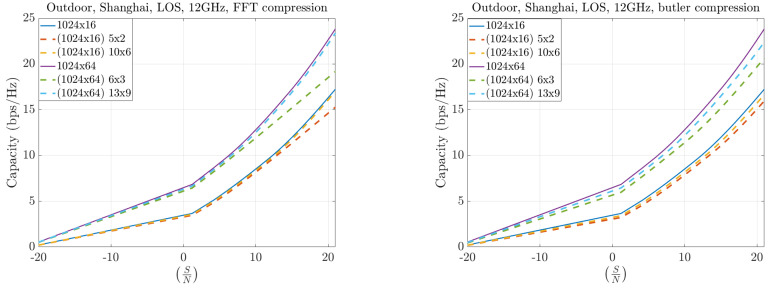
Capacity results at 12 GHz for asymmetric MIMO configurations.

**Figure 20:**
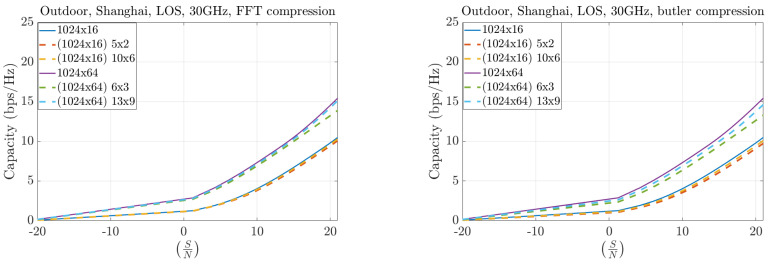
Capacity results at 30 GHz for asymmetric MIMO configurations.

Figure 19 and Figure 20 illustrate the case of asymmetric configurations, where the number of antennas at the base station (BS) greatly exceeds that at the user equipment (UE). In these scenarios, the compression performance remains highly similar between the FFT and Butler matrix approaches. The throughput differences become more pronounced at higher frequencies (30 GHz compared to 12 GHz), reflecting the increased channel sparsity at millimeter-wave bands.

Overall, the results confirm that both methods sustain high spectral efficiency even when the channel matrix is compressed. The Butler matrix thus emerges as a practical alternative to FFT-based processing: it achieves comparable capacity while significantly reducing digital computational load, making it particularly appealing for large-scale implementations.

It should be emphasized that these capacity results correspond to the T-MIMO scenarios considered in [1], which exhibit pronounced angular sparsity at 12 GHz and 30 GHz due to a small number of dominant propagation paths. In channels with reduced sparsity, for example when diffuse scattering or rich, correlated multipath are present, the energy is distributed over a larger number of Butler beams. In such cases, aggressive truncation of the beamspace representation leads to more noticeable capacity losses, and the number of retained beams *m* must be increased to preserve performance, thereby reducing the RF-chain savings. Likewise, imperfect alignment between physical angles of arrival/departure and the discrete DFT grid causes energy leakage into neighboring beams, partially reducing sparsity but not invalidating the method; this effect can be mitigated by using sufficiently dense angular sampling (larger arrays) or oversampled beamspace grids. At sub-6 GHz, where channels are typically less sparse and more diffuse, the relative benefits of angular-domain compression are therefore expected to be smaller than in the T-MIMO and mmWave regimes considered here.

### Extension to Multi-User MIMO Operation

The results in this work focus on a single-user MIMO configuration in order to clearly quantify the impact of angular-domain compression and the differences between FFT- and Butler-based implementations. In practical massive MIMO deployments, however, the base station (BS) typically serves multiple user equipments (UEs) simultaneously. In this subsection we briefly discuss how the proposed architecture can be extended to multi-user (MU-MIMO) operation and how the beam selection mechanism can be adapted in that context.

At the BS side, the two-dimensional Butler matrix implements an approximate unitary transform from the antenna domain to a set of orthogonal or quasi-orthogonal angular beams. In the single-user case, a small subset of these beams is selected to concentrate the channel energy and reduce the number of active RF chains. In the MU-MIMO case, the same beamspace representation can be used as the basis for spatial division multiple access: different users are associated with different angular beams (or groups of beams), and low-dimensional digital precoding is carried out across the selected beams to manage inter-user interference.

A possible downlink procedure is as follows. During a training phase, each UE observes pilots transmitted over a set of beamspace directions and reports (or implicitly reveals through feedback or uplink sounding) its dominant beams. After this step, the BS scheduler selects a collection of users whose dominant beams are sufficiently distinct in angle, following the same design principles as conventional beamspace MU-MIMO. The RF switching fabric is then configured so that the selected beams are connected to a reduced number of RF chains; each RF chain is associated with one or a few beams that are assigned to one or more users. On top of this analog front-end, digital precoding is performed in the reduced-dimensional beamspace in order to perform power allocation and residual interference suppression among the scheduled users.

From a hardware point of view, the architectural modifications required to support MU-MIMO are minimal. The Butler matrix, power-detection network, WTA circuits, and Benes-based switching fabric remain unchanged; what changes is the control logic and the digital baseband. The control logic must now support the simultaneous activation of several sets of beams (one per scheduled user or user group), while the digital baseband must implement a multi-user precoder (e.g., zero-forcing or regularized zero-forcing) in the compressed beamspace. The number of active RF chains is then determined by the total number of beams required to serve the scheduled users, rather than by a single-user compression threshold.

On the UE side, the proposed framework is compatible with both single-antenna UEs and UEs equipped with small antenna arrays. In the single-antenna case, each UE effectively observes a scalar combination of the BS beams and simply reports its strongest downlink beams (or uses uplink pilots to allow the BS to estimate them). In the case of small UE arrays, a compact Butler network can also be employed at the UE to form a small number of angular beams, after which the same dominant-beam selection logic applies. In either case, the dimensionality of the UE-side front-end remains small compared to that of the BS, and the main complexity reduction occurs at the BS.

A full MU-MIMO performance evaluation, including user scheduling strategies and inter-user interference analysis in realistic traffic patterns, is beyond the scope of the present study. Nevertheless, the above discussion shows that the proposed Butler-based beamspace architecture naturally extends to multi-user operation: the analog front-end continues to provide a fixed beamspace basis, while MU-MIMO functionality is realized by selecting appropriate beam sets for each user and applying low-dimensional digital precoding on the corresponding RF chains. This preserves the key advantage of the Butler implementation, namely the reduction in RF-chain count and digital processing load, in both single- and multi-user scenarios.

## 11. Conclusions

Previous work [1] has highlighted the T-MIMO paradigm and the main challenges it introduces for next-generation wireless systems. T-MIMO is expected to play a central role in 6G, particularly at high frequencies, relying on very large antenna arrays at the BS to boost Spectral Efficiency (SE) through spatial diversity and multiplexing. Yet, the full potential of this architecture is constrained by the complexity of CSI acquisition and feedback, which escalates rapidly with array size and bandwidth.

To address this bottleneck, [1] proposed an FFT-based compression scheme. By projecting the channel matrix H into the angular (beamspace) domain using a two-dimensional FFT, the inherent sparsity of the channel was exposed. This sparsity allowed the channel energy to be concentrated in only a few angular bins, enabling compact feedback of the dominant components. Extensive simulations at 10–14 GHz and in the mmW range, across both indoor and outdoor ray-traced environments, demonstrated that this approach significantly reduces feedback overhead while maintaining high CSI fidelity.

Despite these advantages, the FFT-based method faces practical implementation hurdles. Performing large-scale 2D FFTs across hundreds or thousands of antenna ports requires significant digital processing, which increases latency, power consumption, and hardware cost. As antenna arrays scale toward ultra-large dimensions, these challenges undermine the real-time feasibility of the digital-only approach.

To overcome these limitations, this work has developed and analyzed an analog-domain alternative based on Butler matrices. The proposed architecture replaces digital FFTs with 2D Butler networks at both the transmitter and receiver, achieving the same angular decomposition in a passive, near-unitary fashion. After the angular transform, a switching matrix selects only the dominant beams, further reducing the dimensionality before the signals reach the A/D RF chains. This drastically lowers the number of active RF chains and the associated ADC workload, yielding significant power savings.

The system design has been detailed using block diagrams and supported by component-level analysis. Insertion losses of Butler matrices across different technologies (microstrip, SIW, waveguide) were reviewed, and scalable switching strategies were proposed, with Benes networks identified as a practical solution. Power detection and winner-take-all (WTA) circuits were also discussed as enablers for real-time beam selection. Together, these components form an integrated analog front-end that performs channel compression efficiently in hardware.

A complete protocol for system operation was outlined, including training, compression, and transmission phases. Comparative results showed that the Butler-based approach achieves similar capacity performance to FFT-based methods while offering reduced latency and lower power consumption, especially for large arrays. Benchmarking demonstrated that FFT computation time grows significantly with array size and number of subcarriers, whereas Butler-based selection remains nearly constant.

Finally, Table 8 summarizes the trade-offs between FFT and Butler-based compression. While FFT remains compact and flexible in the digital domain, the Butler approach provides a compelling alternative for power- and cost-constrained scenarios, particularly in large-scale mMIMO systems where digital processing overhead becomes prohibitive. These findings indicate that Butler-matrix front-ends are a promising candidate for practical, scalable 6G transceivers, whose potential benefits in latency and energy efficiency are demonstrated here through a combination of realistic component models and channel simulations. Other analog beamspace architectures, such as Blass matrices, Rotman lenses, or hybrid DFT–DFE structures, offer complementary trade-offs in loss, footprint, and bandwidth; our aim is not to claim universal superiority of Butler matrices over these alternatives, but to show that they provide a particularly attractive compromise for compact, low-loss, approximately orthogonal beamspace realizations in planar technologies under the T-MIMO and mmWave scenarios considered. The strongest gains are expected in scenarios with clear angular structure and a limited number of dominant paths, such as the high-frequency T-MIMO and mmWave deployments considered in this work; extending the analysis to richer, less sparse propagation environments and systematically benchmarking alternative analog beamspace solutions within the same framework are interesting directions for future research. A full link-level and over-the-air experimental validation of the proposed architecture is left for future work.

Table 8 summarizes the main trade-offs between three front-end architectures for large-scale MIMO: (i) fully digital FFT-based beamspace processing, (ii) the proposed Butler-matrix beamspace front-end with RF switching and reduced RF-chain count, and (iii) conventional hybrid analog–digital beamforming architectures, both fully connected and sub-connected, as discussed in Section 2. While FFT-based schemes offer the greatest flexibility in the digital domain, their power consumption and latency become critical at ultra-large array sizes. Hybrid beamforming reduces the number of RF chains but requires networks of tunable phase shifters. The Butler-based solution provides a passive, fixed beamspace basis with very low processing latency and a reduced number of RF chains, at the cost of a larger analog footprint and less flexibility in beam shaping.

Other analog beamspace architectures, such as Blass matrices and Rotman lenses, can be regarded as particular instances of the analog or hybrid beamforming category in Table 8, offering complementary trade-offs in loss, footprint, and bandwidth. As discussed in Section 2, Blass networks typically provide greater flexibility in amplitude tapering at the cost of higher loss and larger area, whereas Rotman lenses achieve true-time-delay beamforming with wider bandwidth but bulkier implementations and more complex design.

## Figures and Tables

**Table 1:** Substrate parameters for Butler matrix hybrid couplers.

Parameter	Value
Feed Width	0.333 mm
Feed Length	0.1 mm
Transverse Width	0.333 mm
Longitudinal Width	0.65 mm
Transverse Spacing	6.12 mm
Longitudinal Spacing	6.12 mm
Height	0.5 mm
Ground Plane Distance	1 mm
Relative Permittivity ($\varepsilon_{r}$)	6

**Table 2:** Substrate parameters for Butler matrix phase shifters.

Parameter	Setting 1	Setting 2
Length	3.06 mm	3.06 mm
Width	0.333 mm	0.333 mm
Height	0.5 mm	0.5 mm
Ground Plane Distance	1 mm	1 mm
Relative Permittivity ($\varepsilon_{r}$)	6	6

**Table 3:** Representative excess insertion losses of Butler matrices (10–30 GHz band unless noted).

Reference	Matrix Size	Technology	Freq. (GHz)	Avg. Excess Loss
[16]	4×4	Waveguide (theoretical)	15	0 dB (ideal)
[17]	4×4	SIW (single-layer)	30	∼2 dB (sim.)
[18]	8×8	Microstrip (single-layer)	28	∼2 dB (meas.)
[19]	8×8	SIW (dual-layer)	28–31	∼2 dB (sim.)
[20]	8×8	Microstrip (broadband)	5–6	2–5.6 dB (meas.)
[21]	16×16	Microstrip (multilayer)	9–11	∼2.5 dB (meas.@10)
[22]	16×16	SIW (cascaded 4×4)	28–32	∼1.43 dB (sim.)
[23]	8×8	Waveguide	9.6	<0.4 dB (meas.)

**Table 4:** Estimated depth $L_{\mathrm{depth}}$, array footprint, and aspect ratio for 2D Butler matrices at 10 and 30 GHz ($\varepsilon_{r}=7.5$).

Frequency (GHz)	Array Antenna N×N	$L_{\mathrm{depth}}$ (mm)	Array Size (mm)	Aspect Ratio
10	4×4	26.30	60.00	0.44
10	8×8	39.50	120.00	0.33
10	16×16	52.60	240.00	0.22
10	32×32	65.80	480.00	0.14
30	4×4	8.80	20.00	0.44
30	8×8	13.10	40.00	0.33
30	16×16	17.50	80.00	0.22
30	32×32	21.90	160.00	0.14

**Table 5:** Comparison of RF switch properties from Analog Devices, including frequency coverage, insertion loss, and speed metrics.

Device	Switch Type	Number of Ports	Frequency Range	Insertion Loss (dB)	On/Off Time (ns)	RF Set. Time (ns)
ADRF5238	SPDT	3 (RFC, RF1, RF2)	100 MHz–13 GHz	0.7–1.3	120	220
ADRF5031	SPDT	3 (RFC, RF1, RF2)	9 kHz–20 GHz	0.6–1.05	5000	5300
ADRF5030	SPDT	3 (RFC, RF1, RF2)	100 MHz–20 GHz	0.7–1.2	70	95
ADRF5010	SPST	2 (RF1, RF2)	100 MHz–55 GHz	1.0–2.0	30	50
ADRF5142	SPDT	3 (RFC, RF1, RF2)	8–11 GHz	1.2	60	65
ADRF5048	SP4T	5 (RFC, RF1–RF4)	100 MHz–45 GHz	1.4–4.0	20	60
ADRF5022	SPDT	3 (RFC, RF1, RF2)	100 MHz–45 GHz	1.2–2.3	20	30
ADRF5080	SP8T	9 (RFC, RF1–RF8)	100 MHz–20 GHz	1.3–2.0	55	100
ADRF5054	SP4T	5 (RFC, RF1–RF4)	1–60 GHz	1.7–3.2	25	35

**Table 6:** Performance of representative Winner-Take-All (WTA) circuits reported in the literature.

CMOS Technology	*N* Inputs	Transistor Count	Delay	Resolution	Reference
2 µm (MOSIS)	170	2n	>100 µs	2%	[25]
2 µm (MOSIS)	200	10n	∼300 ns	50 mV	[26]
0.8 µm (AMS)	10	3n	n.p.	1 nA	[27]
0.35 µm (TSMC)	8	12n	∼15 ns	2 nA	[28]
0.25 µm	8	7n	∼50 µs	5 mV	[29]
0.35 µm (AMS)	1024	4n	∼2 µs	10 nA	[30]
0.25 µm (TSMC)	2	3n+4	n.p.	n.p.	[31]
0.5 µm (ON Semi)	4	2n+2	∼1 µs	n.p.	[32]
0.13 µm	3	3n+1	∼50 ns	n.p.	[33]
0.18 µm	2	3n+3	>1 µs	n.p.	[34]
0.18 µm (AMS)	4096	3n+1	<1 µs	n.p.	[35]

**Table 7:** Approximate coherence time $T_{c}$ for different carrier frequencies and user speeds.

Carrier Frequency $f_{c}$ (GHz)	Speed *v* (km/h)	Coherence Time $T_{c}$ (ms)
12	3	≈12.7
12	50	≈0.76
12	120	≈0.32
30	3	≈5.1
30	50	≈0.30
30	120	≈0.13

**Table 8:** Qualitative comparison between fully digital FFT beamspace processing, Butler-matrix front-ends, and hybrid analog–digital beamforming architectures.

Metric	FFT Beamspace (Fully Digital)	Butler-Matrix Beamspace (This Work)	Hybrid Beamforming (Fully/Sub-Connected)
**Power consumption**	High: one RF chain, ADC/DAC and baseband processor per antenna element; large number of high-speed FFTs and matrix operations.	Low to moderate: analog transform reduces the number of active RF chains to the number of selected beams; only a few ADC/DACs and a small digital core are needed.	Moderate: fewer RF chains than fully digital, but per-chain phase shifters, bias networks and control circuits add static and dynamic power.
**Latency**	Processing latency grows with array size and number of frequency channels; 2D FFTs and baseband detection/precoding must be repeated for each update.	Very low: angular transform is passive; latency dominated by power detection, Winner-Take-All selection and RF switching (sub-μs scale).	Digital latency similar to fully digital (baseband precoding/detection) plus additional time to reconfigure phase shifters; reconfiguration speed depends on implementation.
**Spectral efficiency/capacity**	Can closely approach the SVD bound when sufficient angular components are retained; high flexibility in digital precoding and combining.	Comparable to FFT beamspace as long as the same dominant beams are selected; small capacity loss mainly due to analog insertion losses.	Can approach fully digital performance if phase-shifter constraints (constant modulus, quantization) and RF-chain limitations are carefully addressed; some degradation for strongly sub-connected structures.
**Hardware complexity and footprint**	Compact digital implementation but requires one complete RF chain and high-speed data converter per antenna; routing and packaging become challenging for very large arrays.	Requires arrays of hybrids, phase shifters and RF switches; purely passive beamforming network but with a larger physical footprint, especially at lower frequencies.	Intermediate: reduced number of RF chains compared to fully digital, but large networks of variable phase shifters, splitters and combiners increase layout complexity and area.
**Reconfigurability and flexibility**	Highly flexible in the digital domain: arbitrary precoders/combiners and adaptive algorithms are easily implemented in software/firmware.	Beam directions are fixed by the Butler implementation; selection among beams is highly flexible, but beam shapes are essentially static.	Beam directions and patterns can be adjusted continuously (within the resolution of phase shifters); suitable for fine-grained beam steering and null-forming.
**Scalability to ultra-large arrays**	Scaling limited by the cost and power of per-antenna RF chains and baseband processing; requires hardware accelerators for real-time operation.	Well suited to ultra-large arrays: RF-chain count scales with the effective angular support of the channel rather than with the number of elements.	Scales better than fully digital in terms of RF-chain count, but the number of phase shifters and associated control lines grows with the array size, complicating routing and calibration.

## Data Availability

The original contributions presented in this study are included in the article. Further inquiries can be directed to the corresponding author.
